# The application value of serum 25(OH)D3, uric acid, triglyceride, and homeostasis model assessment of insulin resistance in male patients with hyperuricemia combined with hypogonadism

**DOI:** 10.1186/s12902-021-00765-y

**Published:** 2021-05-22

**Authors:** Qun Zhang, Wei Chen, Canqin Yun, Juan Wang

**Affiliations:** Department of Endocrinology, The Sixth Hospital of Wuhan, Affiliated Hospital of Jianghan University, No.168 Hongkong Road, Jianghan District, 430015 Wuhan, Hubei China

**Keywords:** serum 25(OH)D3, serum uric acid, homeostasis model assessment of insulin resistance, triglyceride, male hyperuricemia, hypogonadism, application value

## Abstract

**Background:**

The purpose of this study was to investigate the application value of serum 25(OH)D3, uric acid, triglyceride (TG), and homeostasis model assessment of insulin resistance (HOMA-IR) in male patients with hyperuricemia combined with hypogonadism.

**Methods:**

From August 2018 to August 2020, a total of 198 male patients with primary hyperuricemia were prospectively enrolled in our hospital for inpatient treatment in the department of Metabolism and Endocrinology. They are divided into normal gonadal function group (normal group, *n* = 117) and hypogonadal function group (hypogonadism group, *n* = 81), according to free testosterone (FT) level, International Index of Erectile Function (IIEF-5), and androgen deficiency in the aging male (ADAM) questionnaires. Laboratory indexes were compared between two groups. Multivariate logistic regression was applied to analyze the influencing factors of hypogonadism.

**Results:**

Among the 198 hyperuricemia patients, 40.91 % were hypogonadism. Compared with the normal group, the BMI, waist circumference (WC), and the prevalence of non-alcoholic fatty liver disease (NAFLD), hyperlipidemia (HLP), and obesity (OB) in the hypogonadism group were higher, and the difference was statistically significant (*P* < 0.05, respectively). The levels of fasting plasma glucose (FPG), fasting insulin (FINS), homeostasis model assessment of insulin resistance (HOMA-IR), triacylglycerol (TG), serum uric acid (SUA), alanine transaminase (ALT) of hypogonadism group were higher than those of normal group, while the levels of TT, FT, E2, 25(OH)D3 of hypogonadism group were lower than those of normal group (*P* < 0.05, respectively). Pearson’s linear correlation was used to analyze the correlation between the indicators with significant differences in general data and laboratory indicators and hypogonadism. BMI, WC, HOMA-IR, TG, SUA, TT, FT, 25(OH)D3, E2 were positively correlated with hypogonadism (*r* = 0.556, 0.139, 0.473, 0.143, 0.134, 0.462, 0.419, 0.572, 0.601, *P* = 0.012, 0.027, 0.018, 0.019, 0.028, 0.029, 0.030, 0.009, 0.003, respectively). Taking the above indicators as independent variables and hypogonadism as the dependent variable, logistic regression analysis found that the risk factors for hypogonadism were SUA, WC, BMI, HOMA-IR, TG, TT, FT, E2, and 25(OH) D3.

**Conclusions:**

Serum 25(OH)D3, SUA, HOMA-IR, TG levels were positively correlated with male hyperuricemia patients with hypogonadism. They have important application value in the diagnosis of male hyperuricemia patients with hypogonadism.

**Supplementary Information:**

The online version contains supplementary material available at 10.1186/s12902-021-00765-y.

## Background

In recent years, with the changes in the diet of residents, the incidence of clinical hyperuricemia has not only increased year by year, but also has a younger trend, and is accompanied by a variety of metabolic diseases and cardiovascular and cerebrovascular diseases. Many studies have confirmed that hyperuricemia is a pre-pathological change of kidney stones and gout, and is closely related to the occurrence of metabolic syndrome, coronary heart disease (CHD), atherosclerosis, hypertension and other diseases [1, 2]. Due to the differences in hormone levels, diet, and environmental exposure between men and women, there are significantly more patients with hyperuricemia in men than in women clinically, and hyperuricemia in men may cause hypogonadism [3, 4]. Many studies have confirmed that hyperuricemia has become an important risk factor for male hypogonadism [3, 5].

Hyperuricemia is a metabolic disease caused by increased uric acid synthesis and decreased uric acid excretion. Male hypogonadism is a clinical syndrome caused by androgen deficiency. It is characterized by hyposexuality and decreased gonadal function, which adversely affects the function of multiple organs [6]. In-depth study of the pathophysiological mechanism of hypogonadism in male patients with hyperuricemia, and finding an entry point can be the top priority for the diagnosis of the disease.

Vitamin D is an essential, fat-soluble vitamin in human body, which is closely related to cell proliferation, differentiation, infection and immune system [7]. Uric acid is a marker of metabolic disorders and is associated with stroke, peripheral artery disease, and CHD[8]. HOMA-IR and TG have a certain correlation with metabolic syndrome [9, 10]. However, there is still no conclusion on whether serum 25(OH)D, SUA, HOMA-IR and TG are related to hypogonadism in male patients with hyperuricemia. Based on this, this study will explore the application value of serum 25(OH)D3, SUA, HOMA-IR and TG in male patients with hyperuricemia combined with hypogonadism.

## Methods

### Clinical data

From August 2018 to August 2020, a total of 198 male patients with primary hyperuricemia were prospectively enrolled in our hospital for inpatient treatment in the department of Metabolism and Endocrinology. They are divided into normal gonadal function group (normal group, n = 117) and hypogonadal function group (hypogonadism group, n = 81), according to free testosterone (FT) level, International Index of Erectile Function (IIEF-5), and androgen deficiency in the aging male (ADAM) questionnaires.

The formulation of this research protocol conforms to the relevant requirements of the Declaration of Helsinki of the World Medical Association. The study protocol was approved by Ethics Committee of The Sixth Hospital of Wuhan. All participants signed the informed consent.

### Inclusion and exclusion criteria

The inclusion criteria are as follows: (1) Male patients with primary hyperuricemia, that is, under a normal purine diet, the fasting SUA level was ≥ 420µmol/L tested twice within 2 days [11]. (2) International Index of Erectile Function (IIEF-5) scores [12] < 21 points. (3) Answer “yes” to any 3 questions in androgen deficiency in the aging male (ADAM) questionnaires [13]. (4) The clinical data is complete. (5) The mental is normal.

The exclusion criteria are as follows: (1) Those with severe cardiac insufficiency (NYHA class III-IV). (2) Combined with a malignant disease. (3) At acute stage of gout arthritis. (4) Acute and chronic liver and kidney failure. (5) Combined with infectious and immune diseases. (6) Those who have taken febuxostat, allopurinol, diuretics and other drugs that affect uric acid levels in the past 6 months. (7) Combined with prostate diseases. (8) Those who have been diagnosed with hypogonadism caused by other reasons such as erectile dysfunction or hypopituitarism. (9) Combined with hematological disease (leukemia, aplastic anemia, thrombocytopenia, multiple myeloma). (10) With history of alcohol abuse.

## Methods

### Baseline characteristics collection

The history of gout, hypertension, non-alcoholic fatty liver disease (NAFLD), CHD, type 2 diabetes mellitus (T2DM), alcohol and smoking abuse, medication status were collected. Explain the content and purpose of the IIEF-5 and ADAM questionnaire to the patients, which will be filled out by the patients. The height, weight, waist circumference (WC), and blood pressure were measured.

The IIEF-5 questionnaire includes 5 items, namely, erectile function, orgasmic function, sexual desire, intercourse satisfaction, and overall satisfaction. Each item is scored from 0 to 5, and the higher the score is, the better erectile function is.

The ADAM questionnaire consists of 10 questions, each of which is answered yes or no. If the answer to question 1 or question 7 or any 3 other questions is yes, the result will be judged as positive, otherwise, it will be judged as negative.

### Laboratory examination

The night before the blood sample was collected, the patients were asked to fast for 8–12 h, and sexual life was prohibited. 3-5mL of cubital venous blood was collected at 6:30 am. The sex hormone binding globulin (SHBG), estradiol (E2), follicle-stimulating hormone (FSH), luteinizing hormone (LH), prolactin (PRL), progesterone, testosterone, fasting insulin (FINS), serum creatinine (SCr), blood urea nitrogen (BUN), gamma-glutamyl transferase (GGT), alanine transaminase (ALT), aspartate transaminase (AST), high-density lipoprotein cholesterol (HDL-C), low density lipoprotein cholesterol (LDL-C), triacylglycerol (TG), fasting plasma glucose (FPG), total cholesterol (TC), serum uric acid (SUA), and 25(OH)D_3_.Serum lipids were detected by enzyme method. SHBG, E2, FSH, LH, PRL, progesterone, testosterone, were detected by chemiluminescence method using DXI80O automatic chemiluminescence apparatus (Beckman Coulter, Fullerton, USA). FINS, SCr, BUN, GGT, ALT, AST, HDL-C, LDL-C, TG, TC, SUA, and 25(OH)D_3_ were detected by COULTER CX9 automatic biochemical analyzer (Beckman Coulter, America). HOMA-IR = FPG (mmol/L) × FINS (µU/mL)/ 22.5. Free testosterone (FT) was calculated according to the levels of TT and SHBG through the website: http://www.issam.ch/freetesto.htm.

### Correlation analysis

Correlation analysis was conducted between the laboratory indicators that were statistically different between the two group and hypogonadism. Laboratory indicators that linearly related to hypogonadism was chosen. Multivariate logistic regression analysis was performed between the chosen factors and hypogonadism.

### Statistical analysis

SPSS 23.0 software was used for data statistical analysis. All data were tested for normality and homogeneity of variance. Measurement data was expressed as mean and standard deviation (SD), and analyzed using t-test. Correlation analysis of related laboratory indicators and hypogonadism was used Pearson linear correlation. Multivariate logistic regression was used to analyze the factors of hypogonadism. The level of statistical significance for all the above tests was defined at a probability value of less than 0.05 (*P* < 0.05).

## Results

### Baseline characteristics

Normal group: The age range was 42–68 years old, and the mean age was 56.83 ± 5.82 years. The course of disease was 4–6 years, and the mean course was 5.26 ± 0.36 years. Hypogonadism group: The age range was 42–67 years old, and the mean age was 56.72 ± 5.69 years. The course of disease was 4–7 years, and the mean course was 5.22 ± 0.45 years. Of the 198 male primary hyperuricemia patients, 40.91 % were hypogonadism. Compared with the normal group, the BMI, WC, and the incidence of NAFLD, HLP, OB in the hypogonadism group were higher, with statistical significance (*P* < 0.05, respectively). There was no statistical difference in age, course of disease, systolic blood pressure (SBP), diastolic blood pressure (DBP), and the prevalence of gouty arthritis (GA), CHD, hypertension, T2DM, history of smoking and drinking between the two groups (*P* > 0.05, respectively), see Table 1.

**Table 1:** Baseline characteristics of the two groups

Items	Normal group (*n* = 117)	Hypogonadism group (*n* = 81)	t/χ^2^	*P* value
Age (years, mean ± SD)	56.8 ± 5.82	56.7 ± 5.69	0.132	0.895
Course of disease (years, mean ± SD)	5.26 ± 0.36	5.22 ± 0.45	0.666	0.506
BMI (kg/m^2^, mean ± SD)	25.11 ± 1.33	26.22 ± 1.15	-6.609	<0.001
WC (cm, mean ± SD)	94.01 ± 4.39	96.69 ± 4.23	-4.287	<0.001
SBP (mmHg, mean ± SD)	132 ± 14	135 ± 12	-1.57	0.118
DBP (mmHg, mean ± SD)	84 ± 8	86 ± 9	-1.643	0.102
GA, n ( %)	9 (7.69)	8 (9.88)	0.291	0.59
CHD, n ( %)	7 (5.98)	5 (6.17)	0.003	0.956
Hypertension, n ( %)	60 (51.28)	43 (53.09)	0.062	0.803
T2DM, n ( %))	15 (12.82)	11 (13.58)	0.024	0.877
NAFLD, n ( %)	27 (23.08)	39 (48.15)	13.538	<0.001
HLP, n ( %)	48 (49.57)	58 (59.26)	17.993	<0.001
OB, n ( %)	39 (40.17)	47 (48.15)	11.877	<0.001
Smoking history, n ( %)	23 (19.66)	17 (20.99)	0.052	0.82
Drinking history, n ( %)	36 (30.77)	26 (32.10)	0.039	0.843

*BMI* body mass index; *WC* waist circumference; *SBP* systolic blood pressure; *DBP* diastolic blood pressure; *GA* gouty arthritis; *CHD* coronary heart disease: *T2DM* type 2 diabetes mellitus; *NAFLD* non-alcoholic fatty liver disease; *HLP* hyperlipidaemia; *OB* obesity

### Comparison of laboratory indicators between two groups

The levels of FPG, FINS, HOMA-IR, TG, SUA, and ALT in the hypogonadism group were higher than those in the normal group, and the levels of TT, FT, E2, and 25(OH)D3 in the hypogonadism group were lower than those in the normal group, with statistical differences (*P* < 0.05). Comparison of HbA1c, TC, HDL-C, LDL-C, GGT, AST, BUN, SCr, SHBG, P, PRL, LH, FSH between the two groups showed no statistical difference (*P* > 0.05), see Table 2.

**Table 2:** Comparison of laboratory indicators between two groups

Items	Normal group (*n* = 117)	Hypogonadism group (*n* = 81)	t	*P* value
FPG(mmol/L, mean ± SD)	5.40 ± 1.02	6.52 ± 1.09	-7.386	<0.001
FINS(µU/mL, mean ± SD)	7.29 ± 1.13	10.83 ± 1.29	-20.445	<0.001
HOMA-IR(mean ± SD)	3.03 ± 0.18	3.87 ± 0.21	-30.141	<0.001
HbA_1c_(%, mean ± SD)	7.61 ± 1.23	7.89 ± 1.32	-1.528	0.128
TC(mmol/L, mean ± SD)	4.71 ± 1.03	4.59 ± 1.01	0.812	0.418
TG(mmol/L, mean ± SD)	1.79 ± 0.34	2.58 ± 0.37	-15.503	<0.001
HDL-C(mmol/L, mean ± SD)	2.46 ± 0.36	2.49 ± 0.41	-0.544	0.587
LDL-C(mmol/L, mean ± SD)	2.63 ± 0.24	2.66 ± 0.23	-0.88	0.38
GGT(U/L, mean ± SD)	35.01 ± 1.23	34.89 ± 1.01	0.751	0.454
SUA(µmol/L, mean ± SD)	468.28 ± 36.32	527.39 ± 35.23	-7.927	<0.001
ALT(U/L, mean ± SD)	26.03 ± 2.31	34.01 ± 3.46	-19.465	<0.001
AST(U/L, mean ± SD)	20.18 ± 2.12	20.67 ± 2.14	-1.587	<0.001
BUN(mmol/L, mean ± SD)	7.42 ± 1.23	7.35 ± 1.19	0.399	0.69
SGr(µmol/L, mean ± SD)	99.54 ± 13.83	96.83 ± 14.93	1.312	0.191
TT(nmol/L, mean ± SD)	16.31 ± 2.12	8.73 ± 1.03	29.818	<0.001
SHBG(nmol/L, mean ± SD)	33.53 ± 3.21	32.87 ± 3.18	1.428	0.155
FT(pmol/L, mean ± SD)	306.98 ± 36.38	159.39 ± 23.98	32.003	<0.001
P(nmol/L, mean ± SD)	1.61 ± 0.32	1.57 ± 0.36	0.821	0.413
PRL(mU/L, mean ± SD)	371.93 ± 45.93	375.92 ± 46.69	-0.597	0.551
LH(mU/L, mean ± SD)	8.96 ± 1.38	8.65 ± 1.45	1.522	0.13
FSH(mU/L, mean ± SD)	10.52 ± 2.39	10.87 ± 2.43	-1.006	0.316
E_2_(pmol/L, mean ± SD)	116.93 ± 23.19	98.37 ± 22.23	5.631	<0.001
25(OH)D_3 (_nmol/L, mean ± SD)	39.83 ± 3.21	26.93 ± 3.01	28.514	<0.001

*FPG* fasting plasma glucose; *FINS* fasting insulin; *HOMA-IR* homeostasis model assessment of insulin resistance; *HbA*_*1c*_: hemoglobin A1c; *TC* total cholesterol; *TG* triglyceride; *HDL−C* high−density lipoproteincholesterol; *LDL−C* low−density lipoproteincholesterol; *GGT* gamma−glutamyl transpeptidase; *SUA* serum uric acid; *ALT* alanine transaminase; *AST* aspartate transaminase; *BUN* blood urea nitrogen; *SCr* serum creatinine; *TT* total testosterone; *SHBG* sex hormone binding globulin; *FT* free testosterone; *P* Serum phosphorus; *PRL* prolactin; *LH* luteinizing hormone; *FSH* follicle−stimulating hormone; *E2* estradiol; *25(OH)D3* 25−hydroxyvitamin D−3

### Correlation analysis

Pearson linear correlation was applied to analyze the correlation between BMI, WC, FPG, FINS, HOMA-IR, TG, SUA, ALT, TT, FT, 25(OH)D3, E2 and hypogonadism in baseline data and laboratory indicators. We found that BMI, WC, HOMA-IR, TG, SUA, TT, FT, 25(OH)D3, and E2 were positively correlated with hypogonadism (*r* = 0.556, 0.139, 0.473, 0.143, 0.134, 0.462, 0.419, 0.572, 0.601,*P* = 0.012, 0.027, 0.018, 0.019, 0.028, 0.029, 0.030, 0.009, 0.003, respectively), and FPG, FINS, ALT were not associated with hypogonadism (*r* = 1.101, 1.321, 1.231, *P* = 0.622, 0.691,0.673, respectively).

### Multivariate logistic regression analysis of the factors of hypogonadism

Taking the indicators which were statistically significant between the two groups as independent variables and hypogonadism as the dependent variable. Logistic regression analysis found that the risk factors for hypogonadism were SUA, WC, BMI, HOMA-IR, TG, TT, FT, E2, and 25(OH) D3. See Table 3.

**Table 3:** Multivariate logistic regression analysis of the influencing factors of hypogonadism

Independent variable	β value	Wald χ^2^	*P* value	OR (95 %CI)
BMI	0.167	1.931	< 0.001	1.19(1.030–1.370)
WC	0.126	16.528	0.001	1.22(1.068–1.301)
FPG	0.012	0.021	0.902	1.017(0.849–1.198)
FINS	0.032	0.583	0.512	1.032(0.956–1.132)
HOMA-IR	0.136	4.353	0.029	1.229(1.048–1.236)
TG	0.607	12.573	< 0.001	1.829(1.208–2.561)
SUA	0.019	38.562	< 0.001	1.023(1.016–1.029)
ALT	0.027	0.576	0.432	1.027(0.887–1.123)
TT	0.382	4.347	0.031	1.131(1.050–1.232)
FT	0.379	4.339	0.033	1.142(1.047–1.241)
E_2_	0.621	12.581	< 0.001	1.831(1.331–2.653)
25(OH)D_3_	0.321	4.736	0.009	1.411(1.052–1.763)

*BMI* body mass index; *WC* waist circumference; *FPG* fasting plasma glucose; *FINS *fasting insulin; *HOMA-IR* homeostasis model assessment of insulin resistance; *TG *triglyceride; *SUA* serum uric acid; *ALT* alanine transaminase; *TT* total testosterone; *FT* free testosterone; *E2* estradiol; *25(OH)D3* 25-hydroxyvitamin D-3

## Discussion

Hyperuricemia is a disease of abnormal purine metabolism caused by various causes in the body, resulting in increased uric acid levels in the blood. Relevant studies have confirmed that hyperuricemia is an independent risk factor for chronic kidney disease, kidney stones, cardiovascular and cerebrovascular diseases, atherosclerosis, abnormal glucose metabolism, dyslipidemia, stroke, hypertension and other diseases [1, 2, 14]. The incidence of hyperuricemia in males is significantly higher than that in females, and the prevalence of hyperuricemia in males in China is as high as 12.39 % [15]. A study has confirmed that hyperuricemia causes serious harm to human health, especially metabolic diseases of male, which have a certain impact on male reproductive health, such as decreased testosterone level and erectile dysfunction [16]. In clinic, the decrease of testosterone level may cause male reproductive problems such as gonadal dysfunction, male infertility and physical deterioration. Testosterone is one of the main components of androgens, which is closely related to sperm growth and maturation, reproductive function and maintenance of normal sexual desire.

Total testosterone refers to the sum of the concentrations of protein-bound and unbound testosterone in circulation. It is known that at least four structurally distinct binding proteins can bind testosterone in human circulation, namely, SHBG, human serum albumin (HAS), corticosteroid-binding globulin (CBG), and orosomucoid (ORM). Among these, testosterone is tightly bound to SHBG, and weakly bound to HAS, CBG, and ORM. Only 1–4 % of circulating testosterone is unbound or free. HSBG has been recognized as high-affinity binding protein for testosterone, which is getting much attention [17]. Relevant studies have confirmed that TT has obvious limitations in the diagnosis of hypogonadism, and has no obvious correlation with the questionnaire on symptoms of hypogonadism. TT is mainly used in patients with hypothalamic-pituitary or testicular lesions, but not in men with hypogonadism due to aging [18, 19]. FT is more sensitive to diagnosis of hypogonadism than TT, FT is also significantly correlated with blood lipids, blood glucose, blood pressure. According to the diagnostic criteria for delayed male hypogonadism of the International Society of Andrology (ISA) and the European Urology Association (EAU) [20], FT ≤ 225pmol/L, IIEF-5 score < 21 points, and answering “yes” to any 3 questions in the ADAM questionnaire were considered as the diagnostic criteria for male hypogonadism in this study.

A survey conducted by Deng et al. [21] found that there was no significant difference in WC, BMI, SBP, DBP, HbA1C, TC, TG, LDL-C, HDL-C in male diabetic patients with hypogonadism compared with those of normal gonadal function. Li et al. [22] confirmed that BMI, TG, TC, and FBG of delayed-onset male hypogonadism were statistically different from those of healthy men (*P* < 0.05, respectively), while age and WC were not significantly different from those of healthy men (*P* > 0.05, respectively). The results of this study showed that the age, course of disease, the levels of SBP, DBP, HbA1c, TC, HDL-C, LDL-C, GGT, AST, BUN, SCr, the prevalence of GA, CHD, HBP, T2DM, history of smoking and drinking of hypogonadism group and normal group were comparable (*P* > 0.05). The BMI, WC, and the levels of FPG, FINS, HOMA-IR, TG, SUA, ALT, and the prevalence of NAFLD, HLP, OB in the hypogonadism group were higher than the normal group (*P* < 0.05). The levels of TT, FT, E2, 25(OH)D3 in the hypogonadism group were lower than the normal group (*P* < 0.05). These results suggest that FPG, FINS, HOMA-IR, TG, SUA, ALT levels increase, and TT, FT, E2, 25(OH)D3 levels decrease in male hyperuricemia patients with hypogonadism. But it is not the same as previous studies, which may be caused by different patients and different regions.

Vitamin D is a key factor in the regulation of bone mineralization and calcium homeostasis in both genders. Vitamin D deficiency is widespread worldwide [23]. A recent study has confirmed that vitamin D receptors are widely present in ovary, testis, pituitary, hypothalamus, and the reproductive system is one of the target organs of vitamin D, indicating that vitamin D may be involved in the regulation of gonadal function [24]. Vitamin D needs to be catalyzed by 25-hydroxylase and 1α-hydroxylase in turn in vivo, and converted into the active form of 1,25-hydroxyvitamin D3, thus exerting biological effects. A cross-sectional survey of 3369 community men aged 40–79 conducted by Lee et al. [25] showed that vitamin D deficiency is associated with hypogonadism. SUA is the production of purine metabolism in the constituent nucleic acid and is closely related to metabolic diseases. Cao et al. [26] confirmed that SUA was negatively correlated with serum TT in male T2DM patients, suggesting that SUA was an independent risk factor for low total testosterone. Mukhin et al. [27] found that injecting exogenous androgen into hypogonadism rats could restore the uric acid metabolism level of the rats. HOMA-IR is an index used to evaluate the level of insulin resistance of an individual. In different populations, insulin resistance is negatively correlated with testosterone levels [28]. TG is one of the components of lipids, which are normally in dynamic equilibrium in the body. Haffner et al. [29] confirmed that testosterone was negatively correlated with TG. Svartberg et al. [30] also confirmed that TG level was closely related to testosterone. The results of this study showed that BMI, WC, HOMA-IR, TG, SUA, TT, FT, 25(OH)D3, and E2 were positively correlated with hypogonadism (*P* < 0.05). Logistic regression analysis found that the above indicators are risk factors for hypogonadism, suggesting that male hyperuricemia patients with hypogonadism are closely related to BMI, WC, HOMA-IR, TG, SUA, TT, FT, 25(OH)D3 and E2. The mechanism of hypogonadism caused by low 25(OH)D3 levels in male hyperuricemia patients may be as follows: (1) 25(OH)D3 will increase intracellular Ca^2+^ release, and reduce the mobility of sperm and acrosomal reaction of mature sperm. Vitamin D deficiency will lead to dysregulation of testicular nucleus stromal cells, which will interfere with their normal functions and lead to sperm synthesis dysfunction, thus affecting gonadal function. (2) By regulating the calcium-dependent luteinizing hormone response, luteinizing hormone can cause the secretion of cyclic adenosine-phosphate and the concentration of calcium ions in the testicular stromal cells to produce testosterone. When the level of 25(OH)D3 in the body decreases, the intracellular calcium ion release mediated by vitamin D receptors decreases, and the effect of regulating calcium-dependent luteinizing hormone on adult male reproductive tract, male germ cells and mesenchymal cells decreases. Thus, the synthesis of testosterone in the male reduced, thereby affecting the function of the gonads. In male patients with hyperuricemia, the mechanism of hypogonadism caused by HOMA-IR may be that insulin resistance reduces the level of hypothalamic gonadotropin and affects the level of testosterone; insulin resistance may directly affect the secretion of testosterone in Leydig cells of the testis. In male patients with hyperuricemia, the possible mechanism of TG-induced hypogonadism is that hypertriglyceridemia is often associated with insulin resistance and obesity, and the above two factors are related to hypogonadism. Also, TG is involved in the synthesis, secretion and conversion of active products of testosterone. Increased TG will reduce the synthesis of testosterone, resulting in decreased libido and reduced reproductive capacity. In male patients with hyperuricemia, the main mechanism of hypogonadism caused by SUA is not yet clear. It may be due to the metabolic syndrome affecting the gonadal function, and further mechanism research is still needed.

There are some limitations in this study. First, this is a single-center prospective study, certain selection bias is hard to avoid, and the sample size was limited. Second, this study did not perform subgroup analysis of male hyperuricemia patients with hypogonadism with different levels of BMI, WC, HOMA-IR, TG, SUA, TT, FT, 25(OH)D3, and E2. In addition, the prevalence and treatment outcomes of hypogonadism were not assessed in this study, which need further research.

## Conclusions

Male hyperuricemia patients with hypogonadism are closely related to BMI, WC, HOMA-IR, TG, SUA, TT, FT, 25(OH)D3, E2 levels. SUA, WC, BMI, HOMA-IR, TG, TT, FT, E2, and 25(OH)D3 levels are risk factors for male hyperuricemia patients with hypogonadism. and provide new ideas for the future treatment of hyperuricemia patients with hypogonadism. Regulating the levels of BMI, WC, HOMA-IR, TG, SUA, TT, FT, 25(OH)D3 and E2 in the body may become a new method for the treatment of male hyperuricemia patients with hypogonadism. This provides a new idea for the treatment of hypogonadism in male hyperuricemia patients in the future, and regulating the levels of BMI, WC, HOMA-IR, TG, SUA, TT, FT, 25(OH)D3 and E2 may become a new method for the treatment of this disease.

## Supplementary Information

**Additional file 1:**

## Data Availability

The datasets generated and/or analysed during the current study are not publicly available due to repository at a private clinic but are available from the corresponding author on reasonable request.

## Abbreviations

ADAM: Androgen deficiency in the aging male
ALT: Alanine transaminase
AST: Aspartate transaminase
BUN: Blood urea nitrogen
CHD: Coronary heart disease
DBP: Diastolic blood pressure
E2: Estradiol
FINS: Fasting insulin
FPG: Follicle-stimulating hormone
FSH: Follicle-stimulating hormone
FT: Free testosterone
GA: Gouty arthritis
GGT: Gamma-glutamyl transferase
HDL-C: High-density lipoprotein cholesterol
HLP: Hyperlipidemia
HOMA-IR: Homeostasis model assessment of insulin resistance
IIEF-5: International Index of Erectile Function
LDL-C: Low density lipoprotein cholesterol
LH: Luteinizing hormone
NAFLD: Non-alcoholic fatty liver disease
OB: Obesity
PRL: Prolactin
SBP: Systolic blood pressure
SCr: Serum creatinine
SHBG: Sex hormone binding globulin
SUA: Serum uric acid
TG: Triglyceride
WC: Waist circumference
