# Supplementary material for: The application value of serum 25(OH)D3, uric acid, triglyceride, and homeostasis model assessment of insulin resistance in male patients with hyperuricemia combined with hypogonadism
Source: BMC Endocr Disord. 2021 May 22;21:102. doi: 10.1186/s12902-021-00765-y (PMC8141127; doi:10.1186/s12902-021-00765-y)
Supplement: Supplementary file 1 — Additional file 1: [file 12902_2021_765_MOESM1_ESM.docx]

Androgen Deficiency in the Aging Male (ADAM) Questionnaire

1. Do you have a decrease in libido?

Yes

No

2. Do you have a lack of energy?

Yes

No

3. Do you have a decrease in strength and/or endurance?

Yes

No

4. Have you lost height?

Yes

No

5. Have you noticed a decreased enjoyment of life?

Yes

No

6. Are you sad and/or grumpy?

Yes

No

7. Are your erections less strong?

Yes

No

8. Have you noticed a recent deterioration in your ability to play sports?

Yes

No

9. Are you falling asleep after dinner?

Yes

No

10. Has there been a recent deterioration in your work performance?

Yes

No
